# Predicting response to tVNS in patients with migraine using functional MRI: A voxels-based machine learning analysis

**DOI:** 10.3389/fnins.2022.937453

**Published:** 2022-08-05

**Authors:** Chengwei Fu, Yue Zhang, Yongsong Ye, Xiaoyan Hou, Zeying Wen, Zhaoxian Yan, Wenting Luo, Menghan Feng, Bo Liu

**Affiliations:** ^1^Department of Radiology, The Second Affiliated Hospital of Guangzhou University of Chinese Medicine, Guangzhou, China; ^2^The Second Clinical College, Guangzhou University of Chinese Medicine, Guangzhou, China; ^3^Department of Radiology, The First Affiliated Hospital of Henan University of Chinese Medicine, Zhengzhou, China

**Keywords:** migraine, transcutaneous vagus nerve stimulation, machine learning, functional magnetic resonance imaging, support vector machine (SVM), vagus nerve

## Abstract

**Background:**

Migraine is a common disorder, affecting many patients. However, for one thing, lacking objective biomarkers, misdiagnosis, and missed diagnosis happen occasionally. For another, though transcutaneous vagus nerve stimulation (tVNS) could alleviate migraine symptoms, the individual difference of tVNS efficacy in migraineurs hamper the clinical application of tVNS. Therefore, it is necessary to identify biomarkers to discriminate migraineurs as well as select patients suitable for tVNS treatment.

**Methods:**

A total of 70 patients diagnosed with migraine without aura (MWoA) and 70 matched healthy controls were recruited to complete fMRI scanning. In study 1, the fractional amplitude of low-frequency fluctuation (fALFF) of each voxel was calculated, and the differences between healthy controls and MWoA were compared. Meaningful voxels were extracted as features for discriminating model construction by a support vector machine. The performance of the discriminating model was assessed by accuracy, sensitivity, and specificity. In addition, a mask of these significant brain regions was generated for further analysis. Then, in study 2, 33 of the 70 patients with MWoA in study 1 receiving real tVNS were included to construct the predicting model in the generated mask. Discriminative features of the discriminating model in study 1 were used to predict the reduction of attack frequency after a 4-week tVNS treatment by support vector regression. A correlation coefficient between predicted value and actual value of the reduction of migraine attack frequency was conducted in 33 patients to assess the performance of predicting model after tVNS treatment. We vislized the distribution of the predictive voxels as well as investigated the association between fALFF change (post-per treatment) of predict weight brain regions and clinical outcomes (frequency of migraine attack) in the real group.

**Results:**

A biomarker containing 3,650 features was identified with an accuracy of 79.3%, sensitivity of 78.6%, and specificity of 80.0% (*p* < 0.002). The discriminative features were found in the trigeminal cervical complex/rostral ventromedial medulla (TCC/RVM), thalamus, medial prefrontal cortex (mPFC), and temporal gyrus. Then, 70 of 3,650 discriminative features were identified to predict the reduction of attack frequency after tVNS treatment with a correlation coefficient of 0.36 (*p* = 0.03). The 70 predictive features were involved in TCC/RVM, mPFC, temporal gyrus, middle cingulate cortex (MCC), and insula. The reduction of migraine attack frequency had a positive correlation with right TCC/RVM (r = 0.433, *p* = 0.021), left MCC (r = 0.451, *p* = 0.016), and bilateral mPFC (r = 0.416, *p* = 0.028), and negative with left insula (r = −0.473, *p* = 0.011) and right superior temporal gyrus/middle temporal gyrus (r = −0.684, *p* < 0.001), respectively.

**Conclusions:**

By machine learning, the study proposed two potential biomarkers that could discriminate patients with MWoA and predict the efficacy of tVNS in reducing migraine attack frequency. The pivotal features were mainly located in the TCC/RVM, thalamus, mPFC, and temporal gyrus.

## Introduction

Migraine, affecting approximately 1 billion people, is the second most prevalent neurologic disorder, which imposes socioeconomic burdens and absence of work and study, and can be divided into episodic migraine (attacks that occur ≤ 15 days/month) and chronic migraine (attacks that occur >15 days/month) (Ashina, 2020; Mu et al., 2020). In all subtypes, migraine without aura (MWoA) was the most common one, experienced by the majority of migraineurs (Launer et al., 1999). Currently, the diagnosis of MWoA mainly depends on the International Classification of Headache Disorders (ICHD) (Arnold, 2018), with five criteria including unilateral location with pulsating quality, moderate to severe pain intensity, suffering for 4 to 72 h in one attack, and presence of nausea/vomiting and/or photophobia/phonophobia. Nevertheless, many patients have difficulties in meeting the entire criteria of MWoA in clinical practice (Ozge et al., 2015). Therefore, it is necessary to find an objective as well as an accurate method to diagnose MWoA in the “gray zones.”

In addition, as the pathophysiology remains misty, the pharmacotherapy of migraine is far from satisfactory. Accumulating evidence suggests that transcutaneous vagus nerve stimulation (tVNS) at the external ear can induce anti-nociception. By stimulating the auricular and cervical branches of the vagus nerve non-invasively, migraineurs experienced a significant decrease in the attack frequency and intensity (Straube et al., 2015; Diener et al., 2019; Zhang et al., 2020). Consequently, tVNS has got a Class I recommendation for patients with episodic migraine (Tassorelli et al., 2018; Blech et al., 2020). Moreover, another two studies investigated the mechanism of tVNS in the treatment of migraine and found that tVNS could inhibit the transmission of trigeminal nociception and cortical spreading depression (Cornelison et al., 2020; Morais et al., 2020). Taken together, the above evidence suggested that tVNS should be considered in the clinical practice of migraine. On the other way, despite the effectiveness of tVNS for MWoA, the efficacy varies considerably across different subjects. Therefore, identifying a valid and objective biomarker for treatment response will be of great importance. Intriguingly, emerging functional magnetic resonance imaging (fMRI) has provided an innovative perspective for migraine, greatly contributing to the understanding of its pathophysiology and therapeutics (Ashina et al., 2021). For example, migraineurs exhibited aberrant patterns in the trigeminal cervical complex (TCC), thalamus, medial prefrontal cortex (mPFC), and temporal gyrus (Xue et al., 2013; Wang et al., 2016; Li et al., 2017). Additionally, a significant correlation could be found between migraine pain intensity and disease duration, with the thalamus and mPFC (Coppola et al., 2018; Qin et al., 2020b). For example, functional connectivity of mPFC and thalamus had a negative correlation with pain intensity of migraine. Moreover, studies previously demonstrated nociceptive stimulation would activate TCC and modulate the endogenous pain circuitry which associated the ascending trigeminal spinal-thalamo-cortical pathways with the migraineur's pain sensitivity (Marciszewski et al., 2018; Lim et al., 2021). All of the above studies have revealed a potential central mechanism of migraine, involving TCC, thalamus, mPFC, and temporal gyrus, which could be the targets of migraine treatment.

In terms of these brain regions, our previous studies have investigated the effects of tVNS in treating migraines. For example, the effects of tVNS could be associated with the mPFC and thalamus, which had a negative correlation with migraine attacks days after tVNS treatment (Luo et al., 2020b; Zhang et al., 2021). Furthermore, our recent study also suggested that tVNS would increase functional connectivity between the middle cingulate cortex (MCC) and periaqueductal gray which were involved in descending pain modulation system (DPMS) (Cao et al., 2021). All of those studies indicated that tVNS would treat migraines by modulating these pain-related regions, which reflected the intrinsic characteristics of migraineurs and their relationship with clinical manifestations. Nevertheless, potential biomarkers still have not been applied to migraines. The goals of current studies mainly concentrate on identifying neuroimaging measures related to phenotypic measures, which often does not generalize to novel individuals, thus, it results in inadequate clinical utility (Bisenius et al., 2017; Scheinost et al., 2019).

As a data-driven technique, multivariate pattern analysis (MVPA) plays an important role in analyzing neuroimaging data and is expected to help solve this problem, due to which it is sensitive to the fine-grained spatial discriminative patterns and exploration of inherent multivariate nature from high-dimensional neuroimaging data and also could provide novel insight into the differences between two groups because it allows the identification of features which contribute the most to individual classification or prediction (Khosla et al., 2019; Rocca et al., 2020). Several sensitive and specific neuroimaging potential biomarkers have been explored by machine learning in psychiatric and neurologic diseases, expected to instruct diagnosis and treatment (Yang et al., 2019; Huang et al., 2020; Luo et al., 2020a; Ma et al., 2020; Schneider et al., 2020; Chiarelli et al., 2021). Some previous studies have used machine learning combined with fMRI to identify migraineurs from healthy controls (HCs) with an accuracy of 83.33 to 91.4% (Chong et al., 2017; Tu et al., 2020; Yin et al., 2020; Chen et al., 2021). Furthermore, previous studies have predicted the efficacy of acupuncture for migraines before treatment in individuals, which developed a personalized medicine strategy based on the predictive model (Tu et al., 2020; Yin et al., 2020). Those studies have indicated that the combination of fMRI and machine learning might be used to diagnose specific patients and predict individual responses to clinical therapy.

Thus, the present study aimed to explore the neuroimaging biomarker which can be used to discriminate migraineurs and predict the efficacy of tVNS for migraines. In this study, we selected fractional amplitudes of low-frequency fluctuations (fALFF) as the feature to construct the biomarker, which could reflect the local spontaneous fluctuation of the fMRI BOLD signal (Zou et al., 2008). The advantage of fALFF is that it does not require a prior hypothesis which strengthens test-retest reliability. Moreover, compared with ALFF, fALFF has higher sensitivity and specificity in detecting regional spontaneous brain activity (Zou et al., 2008). In study 1, we calculated the fALFF of each voxel and imported the fALFF value into support vector machine (SVM) to construct the discriminative model which could discriminate migraineurs and healthy controls (HCs). We assessed the performance of model by analyzing the accuracy of the discriminative model. In addition, extracted the mask of discriminative features for further research. In study 2, we used the mask from study 1 and support vector regression (SVR) to construct the predicting model to predict the reduction of migraine attack frequency after tVNS treatment. Finally, we tested the correlation between regions of interest and the reduction of migraine attack frequency.

## Materials and methods

### Participants

MWoA patients with matched healthy controls (HC) were recruited between May 2017 and May 2019. The study was approved by the Institutional Review Board of the Second Affiliation Hospital, Guangzhou University of Chinese Medicine. This study protocol was registered on the Chinese Clinical Trial Registry (ChiCTR-INR-17010559, February 7, 2017, http://www.chictr.org.cn/hvshowproject.aspx?id=11101). Informed consent was obtained from all participants.

This study was an advanced exploration based on our previously published article (Zhang et al., 2021). Thus, eligible criteria and intervention protocol of MWoA patients would not be listed in detail.

In brief, in study 1, patients diagnosed with MWoA by the International Classification of Headache Disorders, the Second Edition (ICHD-2), were included. The patients were asked to fulfill a 4-week (Weeks 1–4) migraine diary including attack frequency, intensity of each attack, and emotion evaluation. Attack frequency was defined as the International Headache Society Clinical Trials Committee recommended (Diener et al., 2020). The intensity of the attack was assessed by a visual analog scale (VAS) of 0 to 100. A higher score meant more severe pain. Patients were asked to record each intensity of head attack using VAS and the average VAS score of each subject was included in the final analysis. Migraine Specific Quality of Life Questionnaire (MSQ) was used to assess the life quality of migraineurs. Self-rating anxiety scale (SAS) and self-rating depression scale (SDS) were used to evaluate emotion. Then, patients and age, sex-matched HCs were required to complete once MRI scanning. We used the demographics and fMRI data of patients and HCs to construct a discriminate model.

In study 2, patients with MWoA were randomly divided into the real group and sham group, receiving a 4-week (Weeks 5–8) treatment according to the treatment protocol. The real tVNS group was applied at the left cymba concha (the true stimulation site), while the sham tVNS group was stimulated on the left tail of the helix. During the treatment, patients were required to complete another 4-week migraine diary as well as MRI scanning as a post-treatment assessment. We constructed predicting model, using the difference in attack frequency between baseline (Weeks 1–4) and post-treatment (Weeks 5–8) of the real group as a label, and discriminative features of the discriminating model generated in study 1 as inputs.

### Demographic and clinical outcomes statistics analysis

Demographic and clinical outcomes were conducted by SPSS 24.0. T-student analysis and Chi-square analysis were used for continuous and counting variables individually. The significance threshold was set to *p* < 0.05 (two-tailed).

### Image acquisition

A 3.0 T Siemens MRI scanner (Siemens MAGNETOM Verio 3.0 T, Erlangen, Germany) was conducted to scan all participants with a 24-channel phased-array head coil. To minimize head movement and scanner noise, foam padding and earplugs were applied. All of them were required to remain motionless, sober with eyes closed, and avoid thinking of anything in particular. All patients participated in the identical functional MRI (fMRI) scanning sessions before and after 4 weeks of treatment in the interval period (If MWoA patients had a headache attack within 48 h before and after scanning, we would make another appointment for fMRI scanning), while HCs completed only one. Resting-state fMRI encompassing the whole brain was acquired with the following parameters: (1) T1-weighted structural images: TR = 1900 ms, TE = 2.27 ms, flip angle = 9°, FOV = 256 × 256 mm, matrix = 256 × 256, and slice thickness = 1.0 mm. (2) Resting-state fMRI images: repetition time (TR) = 2,000 ms, echo time (TE) = 30 ms, field of view (FOV) = 224 × 224 mm, matrix = 64 × 64, flip angle = 90°, slice thickness = 3.5 mm, interslice ga*p* = 0.7 mm, 31 axial slices paralleled, and 240 time points.

### fMRI preprocessing and fALFF analysis

The fMRI data were preprocessed in Data Processing and Analysis for Brain Imaging 3.0 (DPABI 3.0) (Yan et al., 2016). The main steps were as follow: (1) The first 10 volumes were discarded, followed by slice timing and realignment. (2) After head motion correction, structural images were segmented into grey matter, white matter and cerebrospinal fluid. (3) Functional images were coregistered to the structural images. (4) Functional and structural images were standardized into Montreal Neurological Institute (MNI) space. (5) After correcting head motion with Friston 24, linear trending, white matter, and cerebrospinal fluid were conducted. (6) Finally, a 6-mm Gaussian kernel was used to spatially smooth it and a 3-mm voxel resolution was adopted to further analysis.

Before fALFF calculation, the data were filtered by a frequency window of 0.01 to 0.10 HZ. Fast Fourier transform changed the time series to the frequency series. Each voxel computed and averaged the square root of the power spectrum. Then, fALFF was used to calculate the ratio of the power of each frequency at the low-frequency range (0.01–0.08 Hz) to that of the entire frequency range (0–0.25 Hz). Finally, to make the statistics conveniently, the data transformed into z-maps.

### Classification of MWoA and HCs

First, we performed a group-level two-sample *t*-test on fALFF values between HCs and MWoA, with age, sex, and head motion as covariates. Significant differences for fALFF were assessed with a threshold of *p* < 0.05 and false discovery rate (FDR) correction. Features showing significant differences were retained for the subsequent analyses to construct the discriminating model. Second, a leave-one-out cross-validation (LOOCV) was used in the model to avoid the risk of overfitting. Thus, the analyses were unbiased in the sense that the training features were selected independently of each test case. It was used to obtain the best classifier using a linear SVM algorithm combining a feature selection of F-Score. We took all meaningful voxels with the highest ranks to calculate the accuracy, setting the step until incorporating all features. The performance of a classifier was evaluated by accuracy, sensitivity, and specificity. To measure the robustness of the model, a non-parametric permutation test was performed. More specifically, we randomly permuted the labels and repetitively executed the CV procedure 5,000 times. If the accuracy of the classifier on real class labels was more significant than the accuracies of the classifiers trained on randomly relabeled class labels, this classifier was considered to be well-performing. The significance threshold was set to p < 0.05 (two-tailed). After obtaining the best-performing model, we extracted all discriminative features of the model for visualizing the results. Then, we identified brain regions by setting the threshold to > 30% of the maximum weight vector scores for visualizing the results of classification.

### Prediction of the efficacy of tVNS

We still chose LOOCV as the validationmethod. We also regressed sex, age, and headmotion. The prediction model was constructed by support vector regression (SVR) combining with feature select of weight based on the LIBSVM toolbox. The prediction model was trained using the discriminative feature set gained from study 1. The correlation coefficient was calculated to assess the fitting between predictive and actual values. The significance was measured by permutation testing (permutation times = 1,000). The significance threshold was set to *p* < 0.05 (two-tailed). After obtaining the best-performing model, we extracted all predicting features of the model and identified brain regions by visualizing the results of prediction. To investigate the association between fALFF change (post-per treatment) of predict weight brain regions and clinical outcomes (frequency of migraine attack) in the real group, we extracted the average z values of the brain regions. We then performed a partial correlation analysis between the fALFF z value change and the clinical outcomes (frequency of migraine attack), using age, sex, SAS, SDS, and MSQ as covariates. A threshold of *p* < 0.05 false discovery rate (FDR) corrected was applied for multiple comparisons.

**Figure 1:**
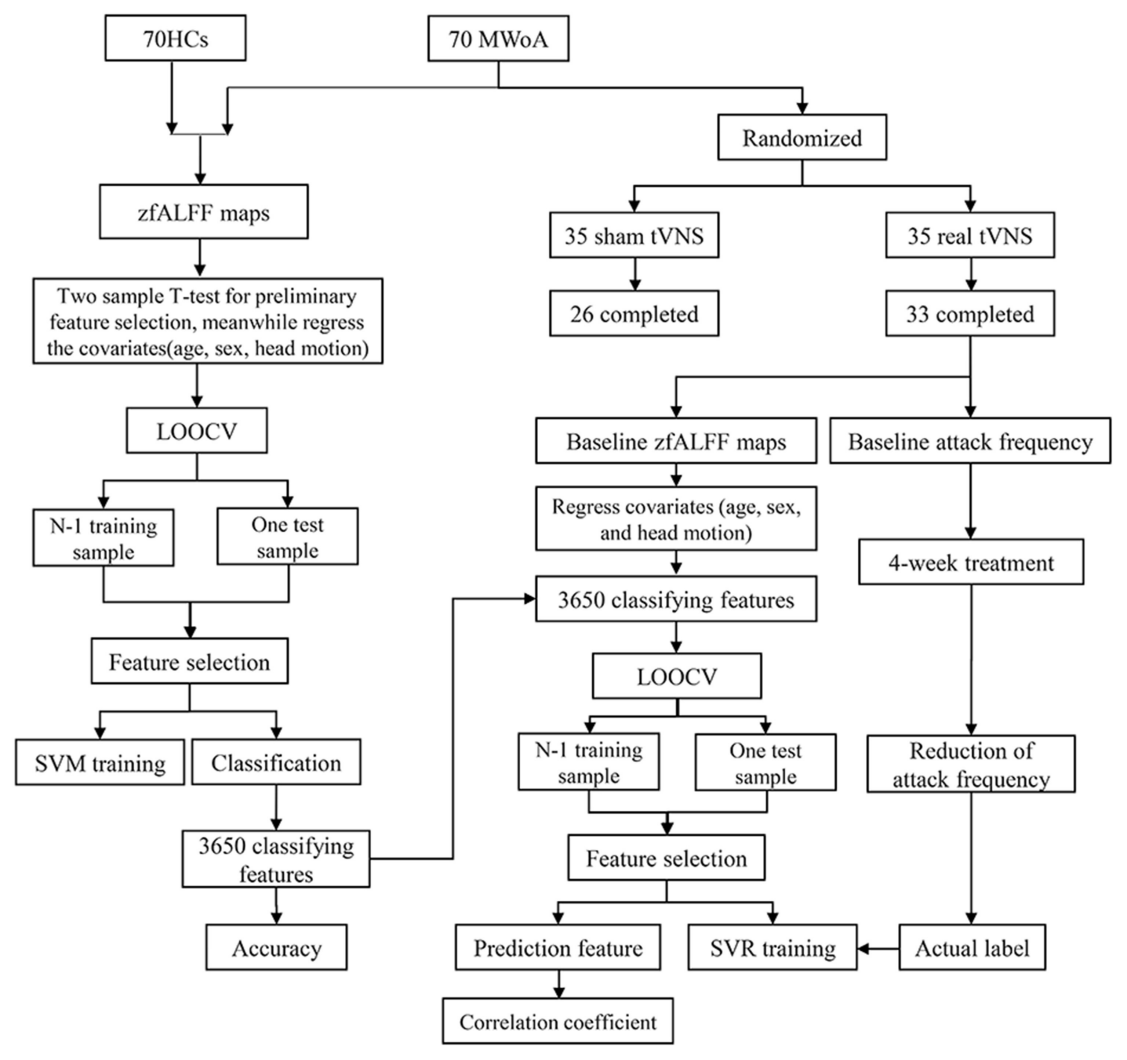
The flow diagram of classification and prediction. A total of 70 HCs and 70 MWOA were used to construct the classification model and the top 3,650 discriminative features could discriminate MWoA with the highest accuracy. Then, the 3,650 discriminative features were used to construct predicting model and 70 predictive features could predict the reduction of migraine attack frequency with the highest correlation coefficient. HCs, healthy controls; MWOA, migraine without aura; LOOCV, leave-one-out cross-validation; SVM, support vector machine; SVR, support vector regression.

See Figure 1 for the flow diagram of classification and prediction.

## Results

### Clinical characteristics

A total of 70 patients with MWoA and 70 HCs participated in the study. They all completed the first scanning fMRI. Nine patients were dropped out in the sham tVNS group (2 for change of residency, 2 for familial dissenting opinion, 2 for time restriction, and 3 for unsatisfied with treatment). Two patients were dropped out in the real tVNS group (1 for time restriction and 1 for unsatisfied with treatment). Consequently, 33 patients with real tVNS and 26 patients with sham tVNS finally completed studies with two times scanning fMRI. Table 1 showed the demographic of participants and migraine characteristics of patients with MWoA. Age and sex between HCs and patients were balanced. After a 4-week treatment, patients in real group indicated a significant reduction in migraine attack frequency (t = 3.341, *p* = 0.002), VAS score of attack intensity (t = 4.614, *p* < 0.001), SAS (t = 4.627, *p* < 0.001), SDS (t = 3.900, *p* < 0.001), and MSQ (t = 6.603, *p* < 0.001).

**Table 1:** Characteristics of each subject group.

**Study 1**				
	**MWoA (*****n** =* **70)**	**HCs (*****n** =* **70)**	**t/**χ^2^	***p*** **value**
Age (years)	30.34 ± 7.20	28.07 ± 6.71	1.93	0.056
Sex (males/females)	16/54	25/45	2.794	0.095
Attack frequency (times/month)	3.81 ± 2.39			
VAS	49.77 ± 15.45			
SAS	43.39 ± 5.64			
SDS	44.88 ± 5.97			
MSQ	57.23 ± 9.94			
**Study 2 (*****n** =* **33)**				
	**Before**	**After**	**t/**χ^2^	***p*** **value**
Age (years)	29.94 ± 6.30			
Sex (males/females)	10/23			
Attack frequency (times/month)	4.0 ± 2.3	2.55 ± 2.25	3.341	0.002
VAS	49.98 ± 14.67	32.23 ± 21.26	4.614	<0.001
SAS	43.30 ± 6.15	40.27 ± 6.98	4.627	<0.001
SDS	43.94 ± 6.14	41.0 ± 6.09	3.900	<0.001
MSQ	57.12 ± 9.68	70.76 ± 10.62	6.063	<0.001

*VAS, visual analog scale; SAS, self-rating anxiety scale; SDS, self-rating depression scale; MSQ, Migraine Specific Quality of Life Questionnaire*.

### Classification results

**Figure 2:**
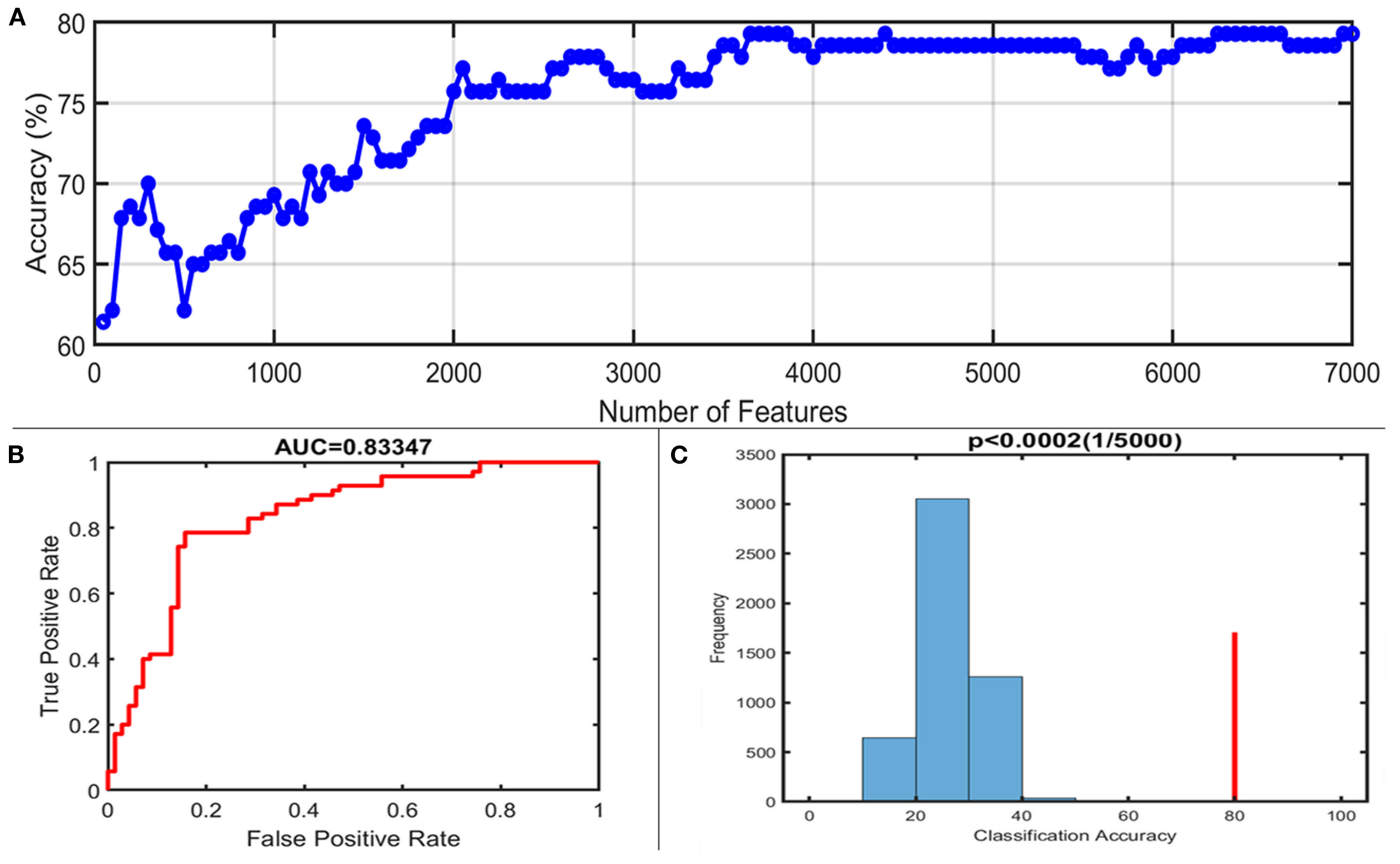
Classification performance of the proposed approach. **(A)** The accuracy of classification with the increased number of features; when including 3,650 discriminative features, the highest accuracy of the classification model is 79.3%. **(B)** Area under the curve of the classification model (AUC = 0.83347) with the highest accuracy. **(C)** The result of the permutation test with the highest accuracy (*p* < 0.0002).

**Figure 3:**
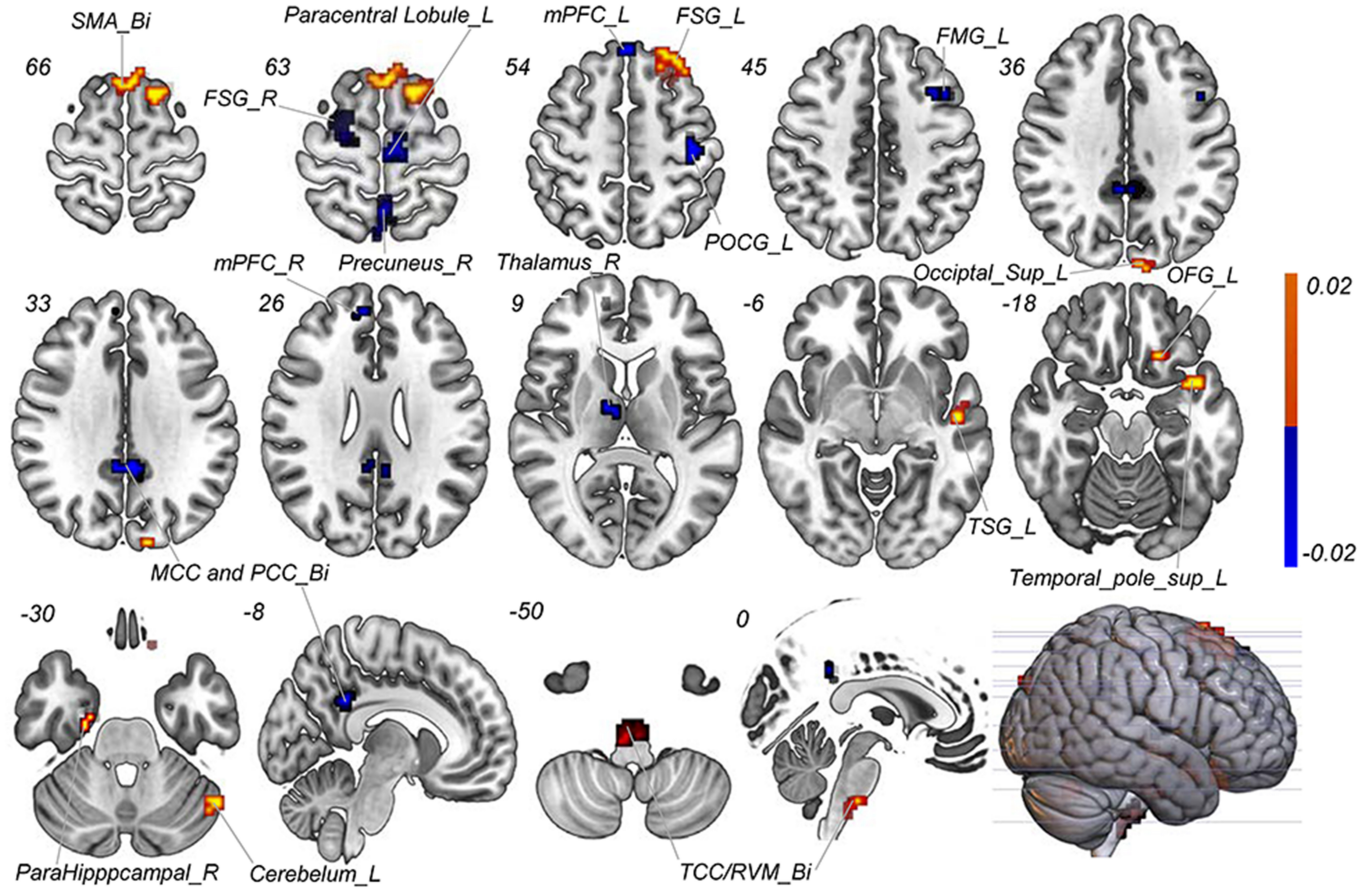
Discriminative features to discriminate MWoA patients and HCs. Red means positive weight and blue means negative weight. The main weight brain areas were located in TCC/RVM, thalamus, PFC, and TSG/TMG. Bi, bilateral; FMG, frontal middle gyrus; FSG, frontal superior gyrus; L, left; MCC, middle cingulate cortex; mPFC, medial prefrontal gyrus; OFG, orbitofrontal gyrus; PCC, post cingulate cortex; POCG, postcentral gyrus; R, right; RVM, rostral ventromedial medulla; SMA, supplementary motor area; Sup, superior; TCC, trigeminal cervical complex; TMG, middle temporal gyrus; TSG, superior temporal gyrus.

With the number of features increasing, the accuracy changed dynamically (Figure 2). The top 3,650 meaningful features showed the best classification ability (79.3% accuracy, 78.6% sensitivity, 80.0% specificity, and 83.35% AUC), which suggested well performance of the result in machine learning. The permutation analysis conducted 5,000 times showed that the classifier with 3,650 meaningful features was superior to the random classifiers (*p* < 0.002). After extracting the discriminative features, we found the classification results of 3650 voxels were enormous and very unfavourable for results demonstrating. According to previous study (Li et al., 2014), we identified brain regions by setting the threshold to >30% of the maximum weight vector scores for visualizing the results of classification (Table 2). The voxels were found in bilateral TCC/rostral ventromedial medulla (TCC/RVM), bilateral mPFC, bilateral MCC, right thalamus, temporal gyrus, right precuneus, and postcentral gyrus, which were involved in trigeminal spinal-thalamo-cortical pathways, default mode network (DMN), auditory network, and DPMS (Figure 3).

**Table 2:** Discriminative features to discriminate MWoA from HCs.

**Weight**	**Cluster voxels**	**Brain region**	**Peak intensity**	**MNI coordinates**		
				**X**	**Y**	**Z**
Postive weight	16	TCC/RVMS_Bi	0.006254	3	−30	−48
	22	Frontal_Sup_L	0.010727	−21	30	57
	13	Temporal_pole_sup_L	0.007674	−39	6	−18
	10	Temporal_Sup_L	0.007724	−51	−15	−6
	18	Supp_Motor_Area_R_L	0.005871	6	21	66
	5	Occipital_Sup_L	0.005292	−12	−93	36
	6	ParaHipppcampal_R	0.005616	21	−12	−30
	11	Cerebelum_Crus1_L	0.008698	−51	−63	−33
Negative weight	8	Thalamus_R	−0.007086	6	−9	9
	24	Cingulum_Mid and Post_Bi	−0.005831	−6	−45	33
	9	Frontal_Sup_Medial_L	−0.008821	−3	42	54
	12	Frontal_Sup_Medial_R	−0.009839	6	51	24
	12	Frontal_ Mid_L	−0.006807	−42	15	45
	9	Frontal_Orb_L	−0.010326	−21	24	−18
	30	Frontal_Sup_R	−0.006637	21	−12	63
	14	Paracentral Lobule_L	−0.008106	−6	−18	66
	12	Postcentral_L	−0.007618	−39	−21	54
	18	Precuneus_L	−0.007034	−3	−69	63

*Bi, bilateral; Inf, inferior; L, left; Mid, middle; Orb, orbit; R, right; RVM, rostral ventromedial medulla; Sup, superior; TCC, trigeminal cervical complex*.

### Prediction of tVNS efficacy

**Figure 4:**
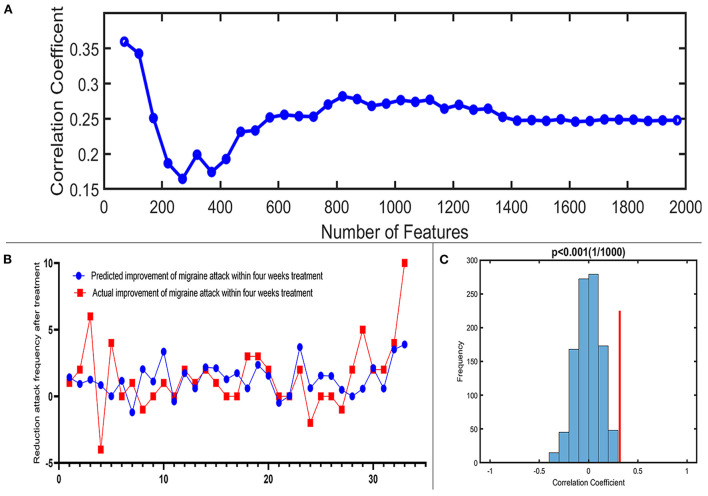
Prediction performance of the proposed approach. **(A)** Line chart reflecting actual label and predictive label; when including 70 predictive features, the highest correlation coefficient is 0.36. **(B)** Correlation coefficient between the actual label and predictive label with the increased number of features in the reduction of migraine attack frequency. **(C)** The result of the permutation test with the highest correlation coefficient (*p* < 0.001).

**Figure 5:**
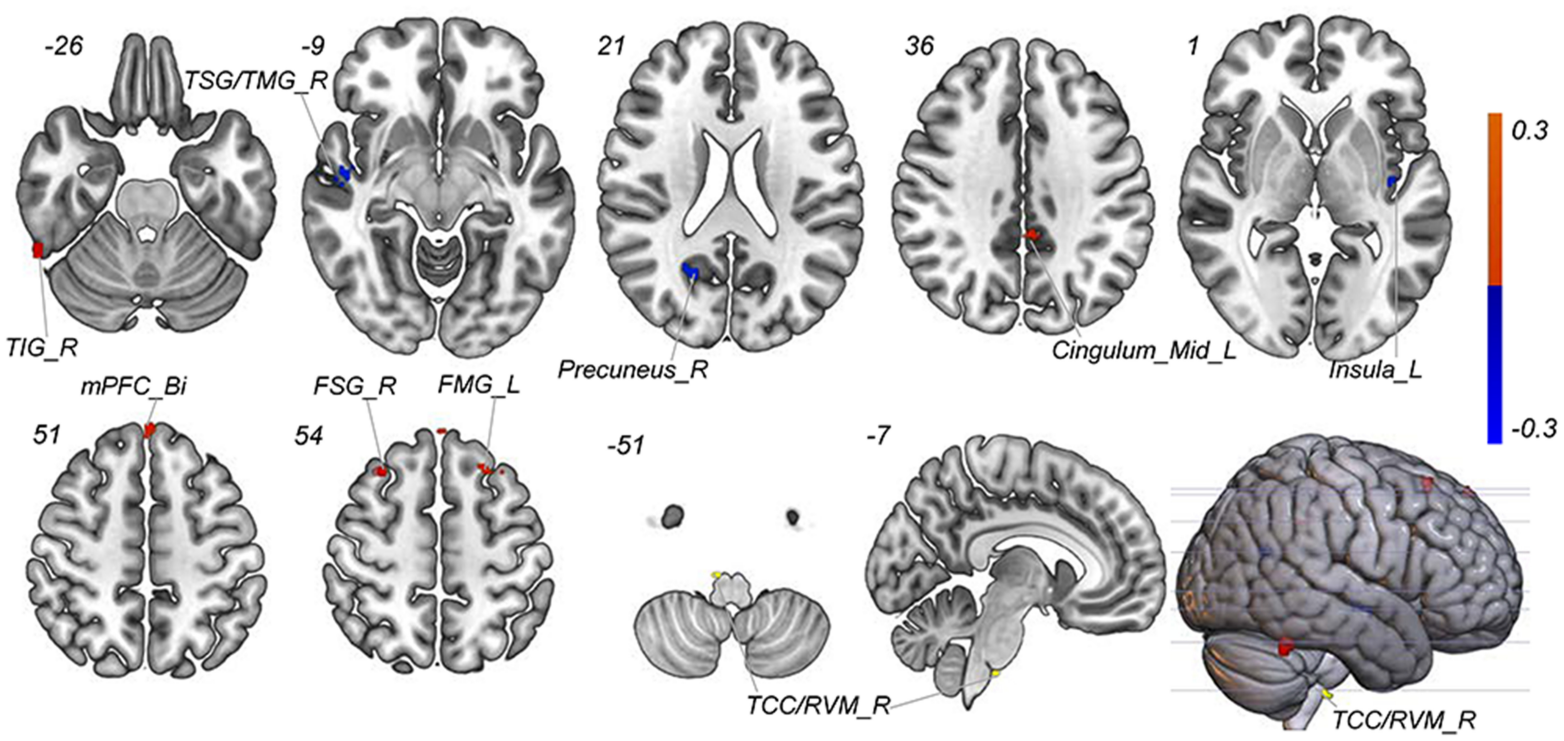
Predictive features to predict the efficacy of tVNS based on the classification model. Red means positive weight and blue means negative weight. The main weight brain areas were located in TCC/RVM, mPFC, TSG, and Insula. Bi, bilateral; FSG, frontal superior gyrus; FMG, frontal middle gyrus; L, left; mPFC, medial prefrontal gyrus; R, right; RVM, rostral ventromedial medulla; TCC, trigeminal cervical complex; TSG, superior temporal gyrus.

**Figure 6:**
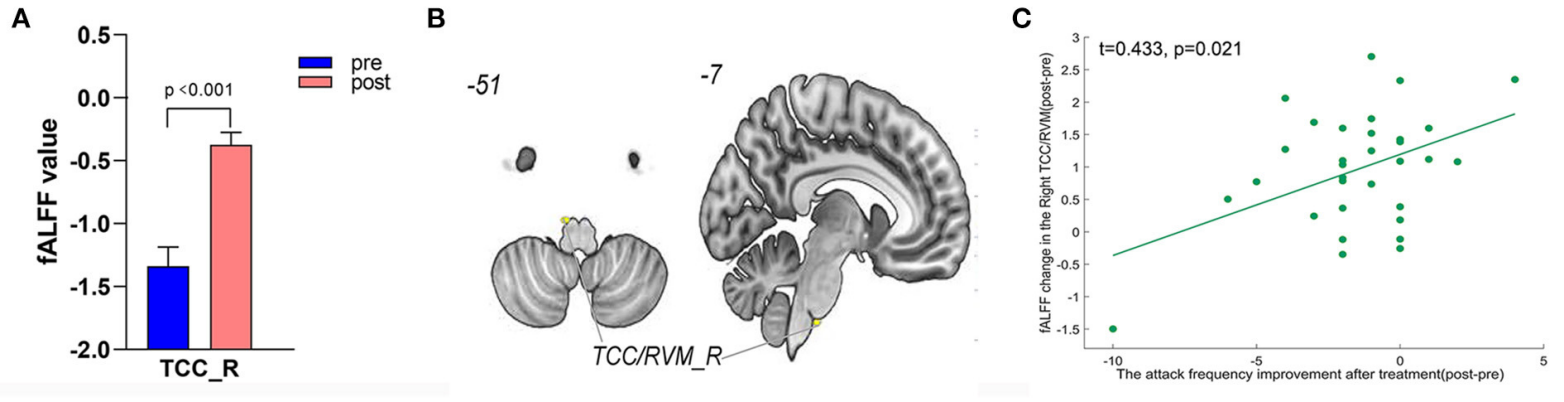
Treatment response of fALFF (mean±sem) and correlation between fALFF changes and migraine frequency changes (FDR corrected). **(A)** Significant increase of fALFF in right TCC/RVM after tVNS treatment. **(B)** Location of TCC/RVM, **(C)** a positive correlation between right TCC and the reduction of migraine attack frequency (r = 0.433, *p* = 0.021). R, right; RVM, rostral ventromedial medulla; TCC, trigeminal cervical complex.

**Figure 7:**
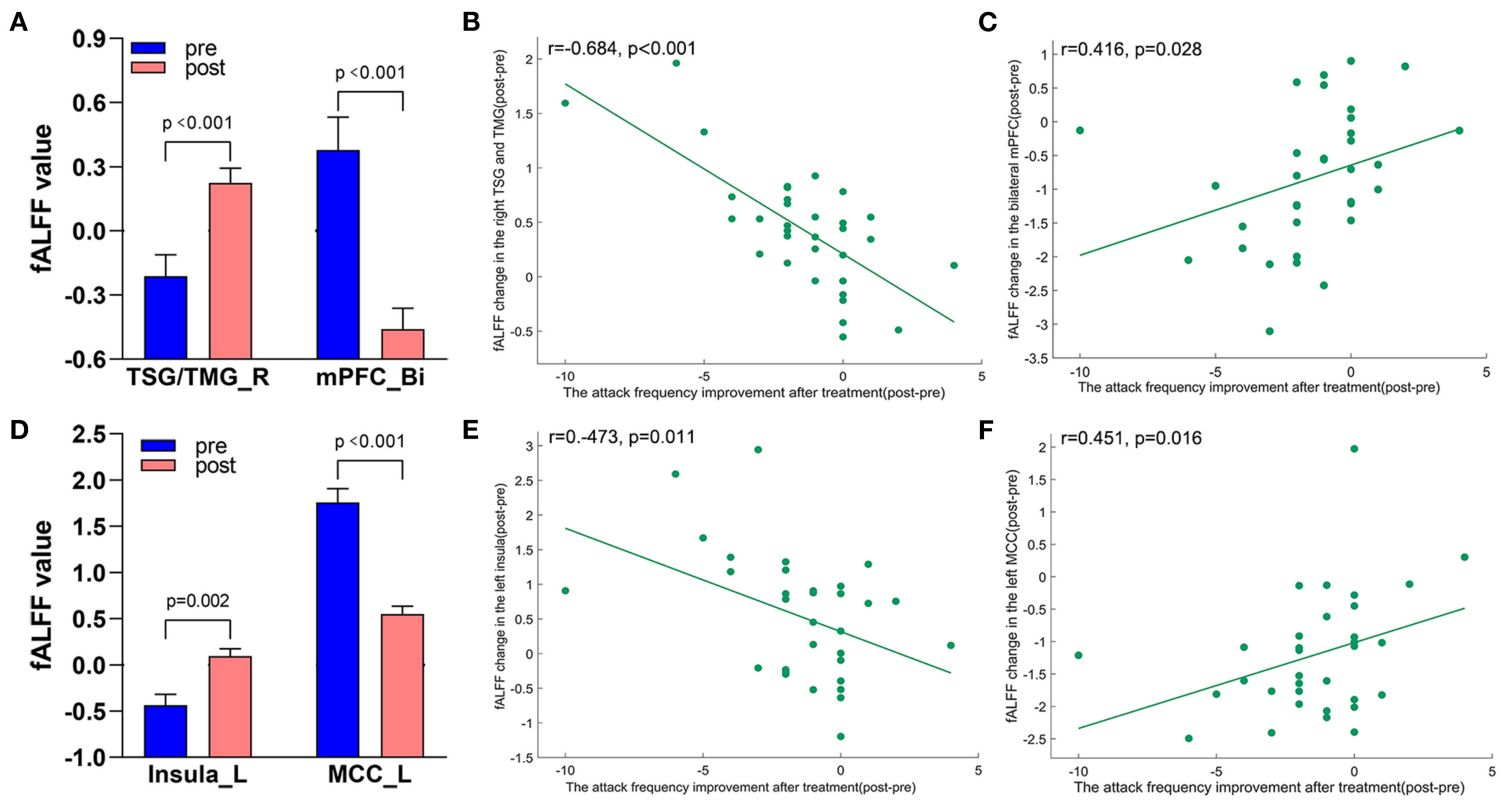
Treatment response of fALFF (mean ± sem) and correlation between fALFF changes and migraine frequency changes (FDR corrected). **(A)** Significant decrease and increase of fALFF in bilateral mPFC and right TSG/TMG after tVNS treatment. **(B)** A negative correlation between right TSG/TMG and the reduction of migraine attack frequency (r = −0.684, *p* < 0.001). **(C)** A positive correlation between bilateral mPFC and the reduction of migraine attack frequency (r = 0.416, *p* = 0.028). **(D)** Significant increase and decrease of fALFF in the left insula and left MCC after tVNS treatment. **(E)** A negative correlation between left Insula and the reduction of migraine attack frequency (r = −0.473, 0.011). **(F)** A positive correlation between left MCC and the reduction of migraine attack frequency (r = 0.451, *p* = 0.016). Bi, bilateral; L, left; MCC, middle cingulate cortex; mPFC, medial prefrontal gyrus; R, right; TMG, middle temporal gyrus; TSG, superior temporal gyrus.

A total of 3,650 discriminative features from study 1 were used to construct the predicting model. We found that 70 of 3,650 discriminative features contributed significantly to predicting the reduction of attacks after a 4-week tVNS treatment (r = 0.36, *p* = 0.03) (Figure 4). After extracting the predictive features, we analyzed the distribution of discriminative voxels (Table 3). The voxels are mainly distributed in TCC/RVM, mPFC, and temporal gyrus (Figure 5). Moreover, a paired *t*-test showed significant fALFF changes in right TCC/RVM, bilateral mPFC, right superior temporal gyrus/middle temporal gyrus (TSG/TMG), left insula, and left MCC. The reduction of migraine attack frequency had a positive correlation with TCC/RVM (r = 0.433, *p* = 0.021), bilateral mPFC (r = 0.419, *p* = 0.029), and left MCC (r = 0.451, *p* = 0.016), and negative with left insula (r = −0.473, *p* = 0.011) and right TSG/TMG (r = −0.684, *p* < 0.001), respectively (Figure 6, Figure 7).

**Table 3:** Predictive features to predict the efficacy of tVNS.

**Weight**	**Cluster voxels**	**Brain region**	**Peak intensity**	**MNI coordinates**		
				**X**	**Y**	**Z**
Positive weight	5	TCC/RVM_R	0.20958	9	−30	−51
	5	Frontal_Sup_Medial_L_R	0.117150	0	63	24
	7	Cingulum_Mid_L	0161300	−3	−42	36
	7	Frontal_Mid_ L	0.189710	−27	24	54
	4	Frontal_Sup_R	0.003670	21	42	51
	8	Temporal_Inf_R	0.067577	57	−51	−30
Negative weight	9	Precuneus_R	−0.109190	21	−63	24
	6	Temporal_Sup_R	−0.222620	51	−12	−6
	3	Insula_L	−0.039590	−42	−12	0

*Inf, inferior; L, left; Mid, middle; Orb, orbitofrontal; R, right; RVM, rostral ventromedial medulla; Sup, superior; TCC, trigeminal cervical complex*.

## Discussion

As an advanced exploration of the previous study (Zhang et al., 2021), by performing complicated machine learning with fMRI, we investigated the potential of spontaneous brain activity in individual diagnosis and treatment in the present study. Our results not only confirmed that the aberrant fALFF patterns served the possibility to be a neuroimaging biomarker with high accuracy (79.3%), sensitivity (78.6%), and specificity (80.0%) in classifying MWoA but also further extended the clinical value of the classification model for predicting the efficacy of tVNS with moderate correlation (r = 0.36). The results indicated that TCC/RVM, MCC, mPFC, and temporal gyrus are the main brain regions in discriminating migraine and predicting the efficacy of tVNS treatment which can be involved in trigeminal spinal-thalamo-cortical pathways, DMN, AN, and DPMS, respectively. Meanwhile, fALFF of the above brain regions had high correlations with the reduction of migraine attack frequency after tVNS treatment. Taken together, our results linked disrupted spontaneous brain activity to migraine and enhanced the comprehension of pathophysiology and treatment of migraine.

In the study, we demonstrated that both TCC/RVM and thalamus could help to identify MWoA which extended previous findings that the thalamus could be used for identifying migraineurs (Chong et al., 2017; Tu et al., 2020). As well-known, TCC and thalamus are the first and second order of trigeminal spinal-thalamo-cortical pathways, cooperating in migraine attacks (Ashina, 2020; Lim et al., 2021). On one hand, TCC is the trigger of sensitization and activation of nociception transmitting nociception from the periphery to the central (Bartsch and Goadsby, 2003; Weir and Cader, 2011; Akerman and Romero-Reyes, 2013; Luz et al., 2019). On the other hand, through comprehensive processing in the selection, amplification, and prioritization, the thalamus could handle the nociceptive inputs from TCC, and then, projected to higher centers inducing pain response (Schwedt et al., 2013; Noseda et al., 2017; Tolner et al., 2019; Tu et al., 2019). The synergistic effect of TCC and thalamus in nociception transmission might be a crucial key to pain response and analgesia.

Consequently, in terms of its important position in the pathophysiology and treatment of migraine, researchers have investigated the aberrant brain alternation of trigeminal spinal-thalamo-cortical pathways in migraineurs. A recent study found a greater BOLD signal variability of the trigeminal spinal-thalamo-cortical pathways in migraineurs than HCs which may amplify nociception processing in migraineurs (Lim et al., 2021). Meanwhile, Meylakh et al. found increasing ALFF of thalamus, TCC/RVM, dorsal pons, and thalamus in migraineurs before an attack (Meylakh et al., 2018). This evidence suggested that TCC and thalamus were coupled to the progress of headache attack events. Moreover, two studies revealed that TCC had a negative correlation with migraine attack intensity, whereas the thalamus had a positive correlation (Hodkinson et al., 2016; Li et al., 2017), revealing a close relationship between the trigeminal spinal-thalamo-cortical pathway and migraine. Further studies suggested that acupuncture, triptans, and tVNS could regulate the disrupted functional connectivity and spontaneous activity within the trigeminal spinal-thalamo-cortical pathways in migraines (Kroger and May, 2015; Moller et al., 2020; Chang et al., 2021; Zhang et al., 2021). And modulation of trigeminal spinal-thalamo-cortical pathways is considered a crucial strategy for the management of migraine (Goadsby et al., 2009). Therefore, it may not be a coincidence that trigeminal spinal-thalamo-cortical pathways could be applied to discriminate migraineurs in our studies.

Another notable finding is that TCC/RVM could predict the efficacy of tVNS, which also had a positive correlation with the reduction of attack frequency. Animal experiments have revealed that tVNS could block the sensitization of TCC by direct and indirect pathways (Lyubashina et al., 2012; Lerman et al., 2019; Sclocco et al., 2019; Vila-Pueyo et al., 2019). Neuroimaging studies provided more straight evidence that tVNS could activate the signal of TCC in participants (Frangos et al., 2015; Frangos and Komisaruk, 2017). All the above studies suggested that tVNS could modulate the function of TCC supporting it as the meaningful brain region in predicting the efficacy of tVNS. Nonetheless, the result did not take the thalamus into the prediction model. But considering its crucial structural connection and synergetic effect with other brain regions of the trigeminal spinal-thalamo-cortical pathways and response to the tVNS (Noseda et al., 2011; Luo et al., 2020b; Lim et al., 2021; Zhang et al., 2021), the effect of thalamus in predicting the efficacy of tVNS in treating migraine could not be neglected.

In addition to the trigeminal spinal-thalamo-cortical pathways, the DMN is another significant brain network in the study consisting of mPFC and precuneus. Consistent with other studies (Tu et al., 2020; Chen et al., 2021), our result showed that DMN could discriminate migraineurs from HCs. The DMN is a network related to individual stressful experiences which could respond to the environment in a predictive manner (Buckner et al., 2008; McEwen and Gianaros, 2011). Nevertheless, the abnormalities in DMN in migraineurs lead to information transfer and multimodal integration dysfunction (Xue et al., 2012; Tessitore et al., 2013; Zhang et al., 2016; Yu et al., 2017). Particularly, researchers have suggested that DMN had a negative correlation with pain intensity, attack frequency, and duration years of migraine (Xue et al., 2012; Gao et al., 2016; Yu et al., 2017; Coppola et al., 2020; Qin et al., 2020a), highlighting the role of DMN in migraine. Additionally, as the integration center, mPFC receives inputs from the thalamus and limbic system, modulating the pain response directly as well as impacting pain management indirectly by regulating emotion and cognition (Ong et al., 2019; Thompson and Neugebauer, 2019; Xu et al., 2019). In short, all the above studies confirmed the critical role of DMN in the pathogenesis of migraine which supported using the DMN to discriminate migraineurs from HCs.

Previous studies have revealed that tVNS could regulate the function of DMN in both physiological and pathological conditions (Kraus et al., 2007; Badran et al., 2018; Wang et al., 2018; Yap et al., 2020; Yakunina and Nam, 2021). Further studies suggested that tVNS would inhibit pain response through the DMN (Usichenko et al., 2017; Guo et al., 2020). What's more, our recent studies demonstrated the changes in the DMN in migraineurs after tVNS treatment (Luo et al., 2020b; Zhang et al., 2021). These findings implicated that DMN might be a potential target of tVNS treatment for migraine. Interestingly, the current study verified that DMN could predict the efficacy of tVNS in migraine management. Especially, fALFF of the mPFC had a positive correlation with the migraine attack frequency. These results expanded our understanding of the important role of the DMN in tVNS treatment for migraine.

Moreover, another finding of the study is that temporal gyrus, MCC, and insula also played important role in discrimination and prediction, whose fALFF value had a correlation with the reduction of attack frequency. These brain regions have two main roles in migraine. For one thing, migraineurs often complain about phonophobia and tinnitus, especially suffering migraine attacks. A reasonable explanation is that MWoA may have a more vulnerable temporal gyrus that is susceptible to external stimuli, causing concomitant symptoms associated with auditory (Langguth et al., 2015; Goadsby et al., 2017). For another, MCC and insula would be responsible for coding pain perception and termination and integrating interoceptive information with emotional salience (Zhao et al., 2020). Acute nociceptive stimuli would consistently activate MCC and insula, affecting a subjective impression of our bodily state, tricking the body into making the wrong decision (Vogt, 2016; Uddin et al., 2017). Although we have provided a robust framework for neural markers in MWoA, there are still several limitations. First, we only recruited subjects suffering from MWoA, lacking comparisons in different subtypes of migraines. Admittedly, a comparison of various subtypes of migraine by machine learning would improve the performance of our model, but considering the morbidity, we thought MWoA should take priority. Second, we only adapted the SVM algorithm without comparing the differences in other algorithms. What needs illustration is that SVM is a growing popularity algorithm for its relative simplicity within the neuroimaging community (Campbell et al., 2020). Third, although we did not perform the sample size estimation, we determined it according to the previously published similar article (Yin et al., 2020). Further larger scale study will be conducted to enhance the data reliability (Page 18, line 431). Finally, it is a single-center study without external validation. Thus, further multi-center research should be carried out to verify the repeatability and generalization of the models.

## Conclusion

In summary, the study preliminarily demonstrated that fALFF features (infra-slow oscillations) at baseline have good potential for classifying the MwoA with the HCs and predicting the individualized treatment response of tVNS. And we provided a pattern for selecting patients to respond well to tVNS for migraine which could optimize the allocation of medical resources. TCC/RVM, thalamus, mPFC, and temporal gyrus are the potential targets both in the classification and prediction model.

## Data availability statement

The original contributions presented in the study are included in the article/supplementary material, further inquiries can be directed to the corresponding author/s.

## Ethics statement

The studies involving human participants were reviewed and approved by Guangdong Provincial Hospital of Chinese Medicine. The patients/participants provided their written informed consent to participate in this study.

## Author contributions

CF: investigation, conceptualization, and writing—original draft. YZ: conceptualization, methodology, formal analysis, and writing—original draft. YY: data curation, supervision, and project administration. XH: methodology and formal analysis. ZW: software, writing—review, and editing. ZY: resources and supervision. WL: data curation and supervision. MF: investigation and data curation. BL: conceptualization, supervision, project administration, and writing—review and editing. All authors approval of the version of the manuscript to be published.

## Funding

This study was supported by the Guangzhou Science and Technology Basic Research Projects (202102010260) and the Traditional Chinese Medicine Science and Technology Project of Guangdong Hospital of Traditional Chinese Medicine (YN2020MS09).

## Conflict of interest

The authors declare that the research was conducted in the absence of any commercial or financial relationships that could be construed as a potential conflict of interest.

## Publisher's note

All claims expressed in this article are solely those of the authors and do not necessarily represent those of their affiliated organizations, or those of the publisher, the editors and the reviewers. Any product that may be evaluated in this article, or claim that may be made by its manufacturer, is not guaranteed or endorsed by the publisher.
